# Supplementary material for: The lysosomal transporter TAPL has a dual role as peptide translocator and phosphatidylserine floppase
Source: Nat Commun. 2022 Oct 4;13:5851. doi: 10.1038/s41467-022-33593-2 (PMC9532399; doi:10.1038/s41467-022-33593-2)
Supplement: Supplementary file 1 — Supplementary Information [file 41467_2022_33593_MOESM1_ESM.pdf]

Supplementary Information

for

The lysosomal transporter TAPL has a dual role as peptide translocator and phosphatidylserine floppase

Jun Gyou Park[1], Songwon Kim[1], Eunhong Jang[1], Seung Hun Choi[1], Hyunsu Han[1], Ji Won Kim[2], Seulgi Ju[1], Da Sol Min[1] & Mi Sun Jin[1,*]

Correspondence to: misunjin@gist.ac.kr

```
                                                TMD0
hTAPL      1  MRLWKAVVVTLAFMSVDICVTTAIYVFSHLDRSLLEDIRHFNIFDSVLDLWAACLYRSCL  60
mTAPL      1  MRLWKAVVVTLAFVSTDVGVTTAIYAFSHLDRSLLEDIRHFNIFDSVLDLWAACLYRSCL  60
              *************:*.*: ******.**********************************

hTAPL     61  LLGATIGVAKNSALGPRRLRASWLVITLVCLFVGIYAMVKLLLFSEVRRPIRDPWFWALF  120
mTAPL     61  LLGATIGVAKNSALGPRRLRASWLVITLVCLFVGIYAMAKLLLFSEVRRPIRDPWFWALF  120
              **************************************.*********************

                                                                  elbow
hTAPL    121  VWTYISLGASFLLWWLLSTVRPGTQALEPGAATEAEGFPGSGRPPPEQASGATLQKLLSY  180
mTAPL    121  VWTYISLAASFLLWGLLATVRPDAEALEPGN----EGFHGEGGAPAEQASGATLQKLLSY  176
              *******.****** **:****.::*****     *** *.*  * **************

                                   TM5
hTAPL    181  TKPDVAFLVAASFFLIVAALGETFLPYYTGRAIDGIVIQKSMDQFSTAVVIVCLLAIGSS  240
mTAPL    177  TKPDVAFLVAASFFLIVAALGETFLPYYTGRAIDSIVIQKSMDQFTTAVVVVCLLAIGSS  236
              **********************************.**********:****:*********

                        TM6                     ICH1           TM7
hTAPL    241  FAAGIRGGIFTLIFARLNIRLRNCLFRSLVSQETSFFDENRTGDLISRLTSDTTMVSDLV  300
mTAPL    237  LAAGIRGGIFTLVFARLNIRLRNCLFRSLVSQETSFFDENRTGDLISRLTSDTTMVSDLV  296
              :***********:***********************************************

                                                  TM8a
hTAPL    301  SQNINVFLRNTVKVTGVVVFMFSLSWQLSLVTFMGFPIIMMVSNIYGKYYKRLSKEVQNA  360
mTAPL    297  SQNINIFLRNTVKVTGVVVFMFSLSWQLSLVTFMGFPIIMMVSNIYGKYYKRLSKEVQSA  356
              *****:****************************************************.*

                   TM8b        ICH2                     TM9
hTAPL    361  LARASNTAEETISAMKTVRSFANEEEEAEVYLRKLQQVYKLNRKEAAAYMYYVWGSGLTL  420
mTAPL    357  LARASTTAEETISAMKTVRSFANEEEEAEVFLRKLQQVYKLNRKEAAAYMSYVWGSGLTL  416
              *****.************************:******************* *********

                                             TM10a                TM10b
hTAPL    421  LVVQVSILYYGGHLVISGQMTSGNLIAFIIYEFVLGDCMESVGSVYSGLMQGVGAAEKVF  480
mTAPL    417  LVVQVSILYYGGHLVISGQMSSGNLIAFIIYEFVLGDCMESVGSVYSGLMQGVGAAEKVF  476
              ********************:***************************************

                                  NBD           A-loop
hTAPL    481  EFIDRQPTMVHDGSLAPDHLEGRVDFENVTFTYRTRPHTQVLQNVSFSLSPGKVTALVGP  540
mTAPL    477  EFIDRQPTMVHDGSLAPDHLEGRVDFENVTFTYRTRPHTQVLQNVSFSLSPGKVTALVGP  536
              ************************************************************

             Walker A                                       Q-loop
hTAPL    541  SGSGKSSCVNILENFYPLEGGRVLLDGKPISAYDHKYLHRVISLVSQEPVLFARSITDNI  600
mTAPL    537  SGSGKSSCVNILENFYPLQGGRVLLDGKPIGAYDHKYLHRVISLVSQEPVLFARSITDNI  596
              ******************:***********.*****************************

                                                        Signature motif
hTAPL    601  SYGLPTVPFEMVVEAAQKANAHGFIMELQDGYSTETGEKGAQLSGGQKQRVAMARALVRN  660
mTAPL    597  SYGLPTVPFEMVVEAAQKANAHGFIMELQDGYSTETGEKGAQLSGGQKQRVAMARALVRN  656
              ************************************************************

               Walker B  D-loop                      H-motif
hTAPL    661  PPVLILDEATSALDAESEYLIQQAIHGNLQKHTVLIIAHRLSTVEHAHLIVVLDKGRVVQ  720
mTAPL    657  PPVLILDEATSALDAESEYLIQQAIHGNLQRHTVLIIAHRLSTVERAHLIVVLDKGRVVQ  716
              ******************************:**************:**************

hTAPL    721  QGTHQQLLAQGGLYAKLVQRQMLGLQPAADFTAGHNEPVANGSHKA                766
mTAPL    717  QGTHQQLLAQGGLYAKLVQRQMLGLEHPLDYTASHKEPPSNTEHKA                762
              *************************:   *:**.*:** :* .***
```

Supplementary Figure 1. Sequence alignment of human and mouse TAPL. The secondary structure indicated (above) refers to mouse TAPL. The N-terminal TMD0 sequence of mouse TAPL (1-142) is shown in grey. The conserved NBD motifs are indicated in light orange (A-loop), yellow (Walker A), blue (Q-Loop), red (Signature motif), green (Walker B), deep blue (D-loop) and purple (H-motif). The Genebank accession numbers for human and mouse TAPL proteins are AB045381.2 [https://www.ncbi.nlm.nih.gov/nuccore/AB045381.2] and AB045382.2 [https://www.ncbi.nlm.nih.gov/nuccore/AB045382.2], respectively.

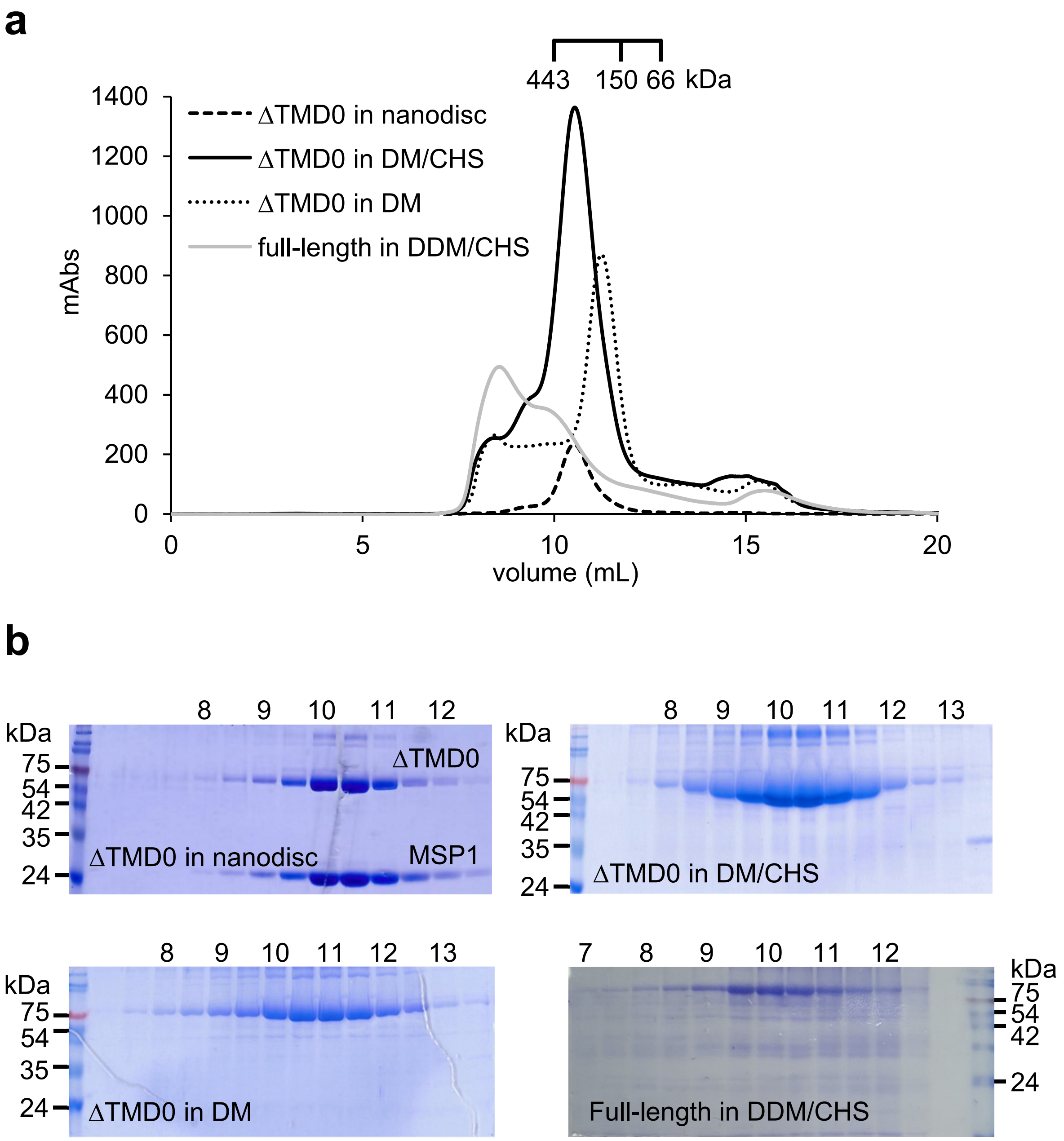

Supplementary Figure 2. Size exclusion chromatography of the full-length and core region of mouse TAPL. (a-b) Proteins purified using nanodiscs made of *E.coli* polar lipids or detergent micelles with and without CHS were monitored by size exclusion chromatography (a) and by SDS-PAGE (b). The number at the top of SDS-PAGE gel represents the elution volume (in milliliters) examined. The gel is one representative result from at least three independent experiments. Source data is provided as source data file.

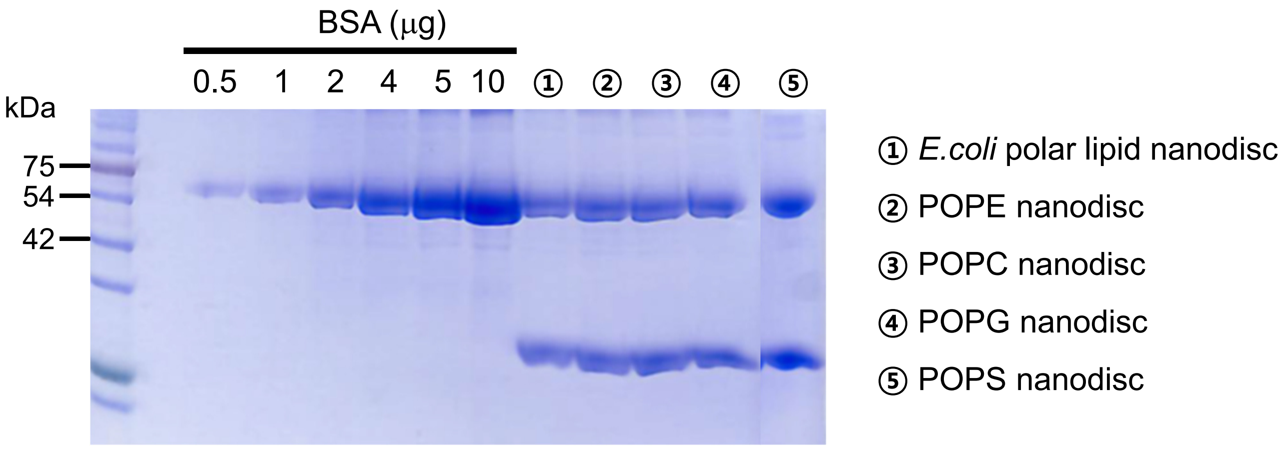

Supplementary Figue 3. SDS-PAGE analysis for measuring ΔTMD0 in nanodiscs. BSA (0.5-10 μg) and ΔTMD0 in nanodiscs (2 μL) were electrophoresed in a 10% SDS polyacrylamide gel. The concentration of nanodisc-reconstituted ΔTMD0 was measured using a BSA standard curve. The SDS-PAGE results show that ~2 μg of protein was reconstituted in each nanodisc. The last lane of sample 5 (POPS nanodisc) was derived from a different SDS-PAGE gel. The gel is one representative result from at least two independent experiments. Source data is provided as source data file.

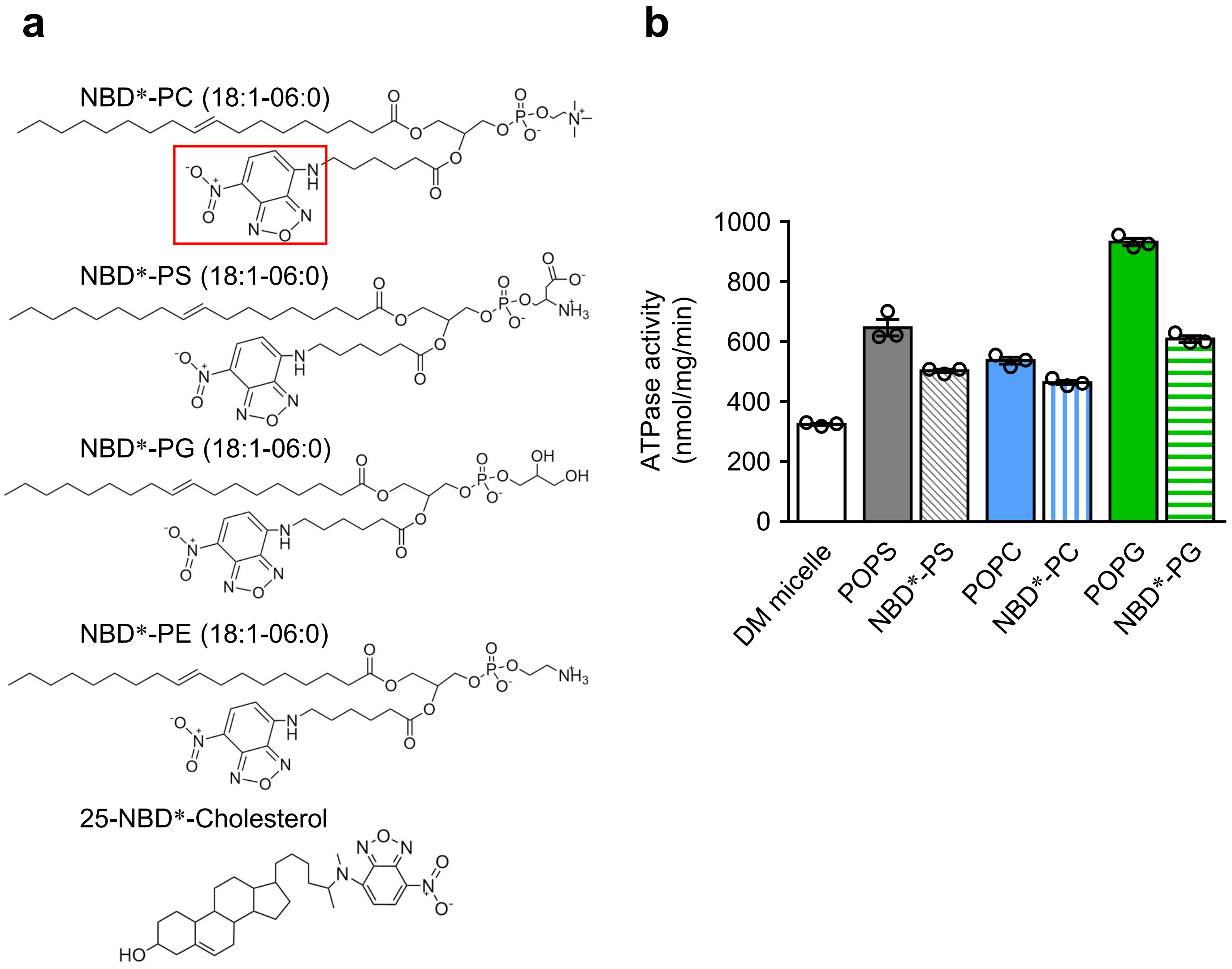

Supplementary Figure 4. Attachment of the NBD* chromophore to the acyl chain of phospholipids has no significant effect on ΔTMD0 ATPase activity. (a) Chemical structures of various NBD*-labeled lipids used in this study. The NBD* group is indicated by the red box. (b) Effect of NBD*-conjugation on the ATPase activity of DM-purified ΔTMD0. At saturating concentrations (250 µM), regardless of NBD* labelling, all lipid substrates tested stimulated the ATPase activity of ΔTMD0 to similar levels. Each point is the mean of three replicate experiments using the same preparation. Error bars represent SEM (± standard error of the means). Source data is provided as source data file.

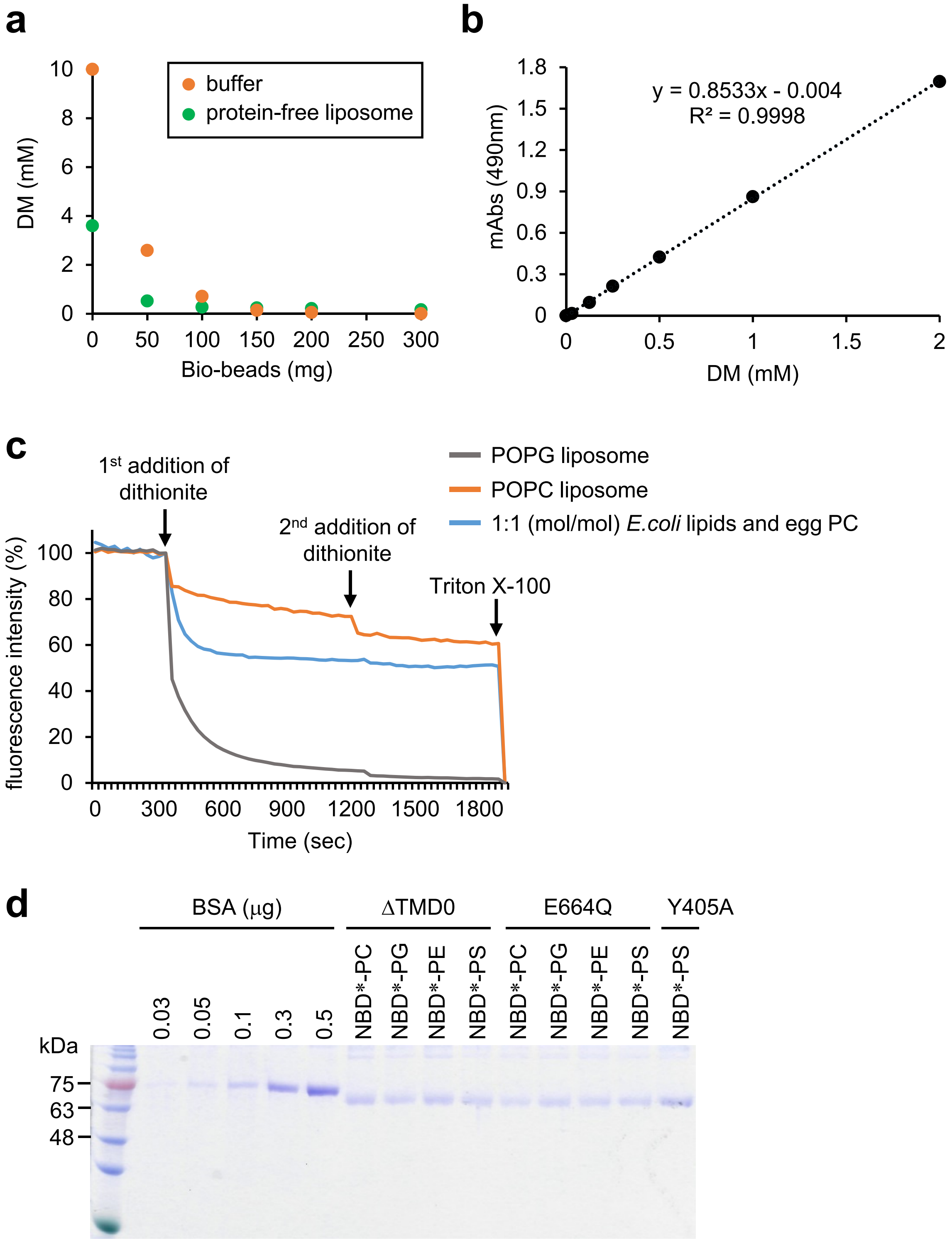

Supplementary Figure 5. Preparation of non-leaky proteoliposomes for accurate measurement of floppase activity. (a) Adsorption of DM detergent by Bio-Beads SM-2 in the buffer (orange) and protein-free liposome system (green). Detergent concentration was quantified by colorimetric assay to determine the amount of Bio-Beads required to remove all detergent. See also Methods. Source data is provided as source data file. (b) Standard calibration curve for quantification of residual detergent after treatment of Bio-Beads. Source data is provided as source data file. (c) Dithionite permeation assay. Screening of various protein-free liposomes containing 0.5 (mol/mol)% NBD*-PS shows that even after two dithionite treatments the fluorescence baseline does not decrease in liposomes composed of a 1:1 (mol/mol) mixture of E. coli lipids and egg PC (blue), whereas it does decrease in liposomes formed by POPC (orange) or POPG (grey). Source data is provided as source data file. (d) SDS-PAGE analysis of proteoliposomes. The amounts of protein in liposomes were calculated using BSA calibration (0.03-0.5 μg). The SDS-PAGE results show that ~0.2 μg of protein was reconstituted in each liposome, resulting in a final lipid-to-protein ratio of ~118:1 (w/w). The gel is one representative result from at least two independent experiments. Source data is provided as source data file.

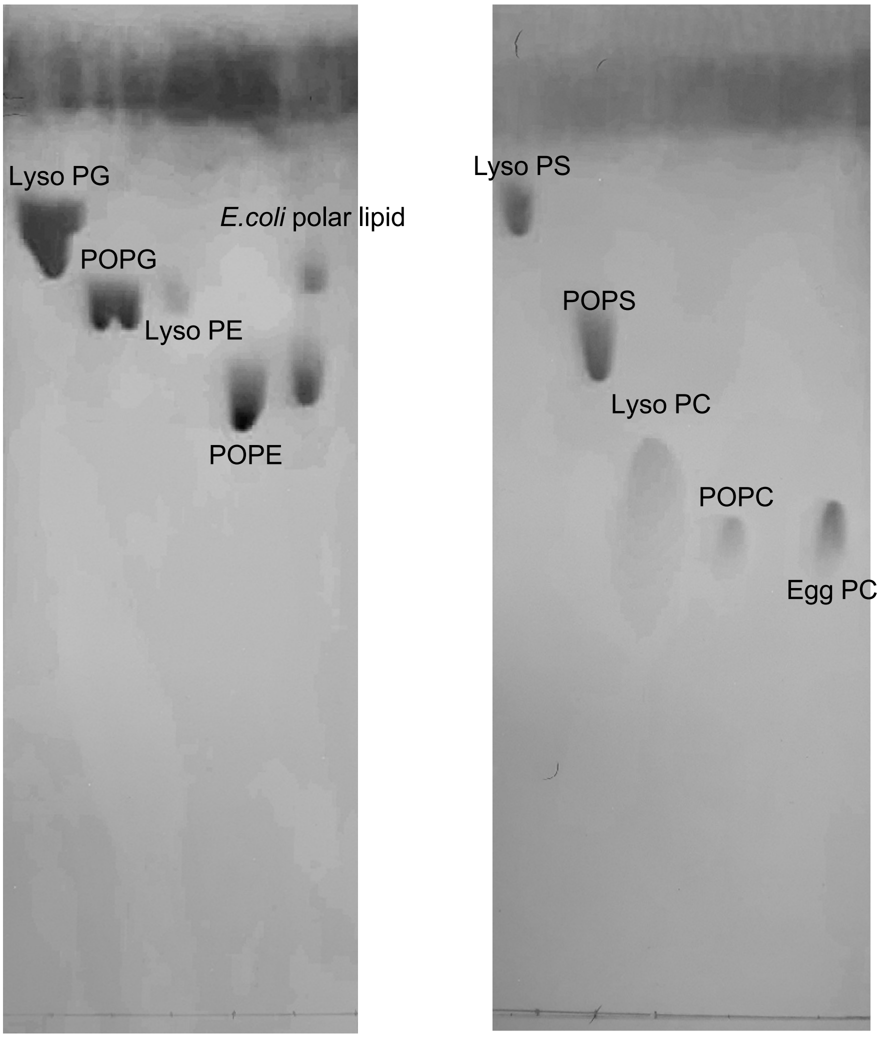

Supplementary Figure 6. Thin-layer chromatography (TLC) of natural phospholipids and their lyso-forms. The TLC separation of phospholipids used in this study and their lyso-forms is shown. The solvent system used was chloroform/methanol/ammonia/water (5:3:0.3:0.15, v/v/v/v). The plate was stained with potassium permanganate solution. Source data is provided as source data file.

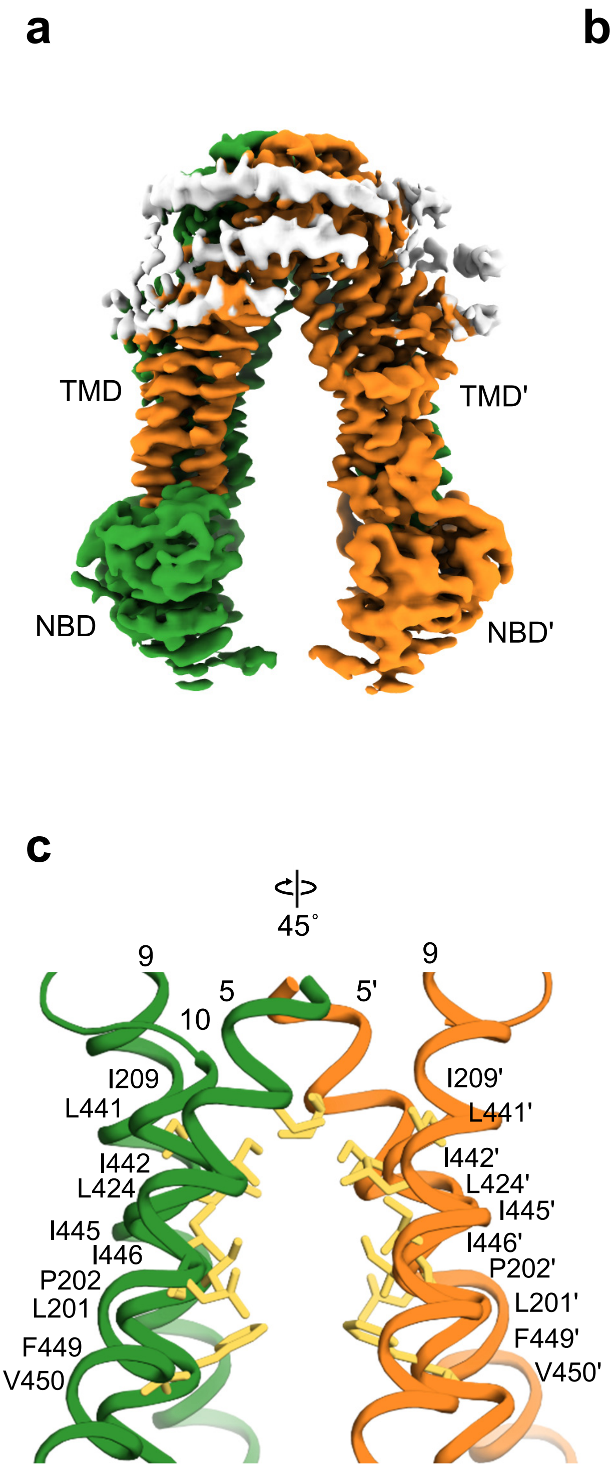

Supplementary Figure 7. Structure of inward-facing ΔTMD0. (a) Cryo-EM 3D reconstruction of inward-facing ΔTMD0. (b) Cartoon representation of the ΔTMD0 monomer. (c) The shape of the upper cavity. The residues lining the upper cavity are shown as yellow sticks.

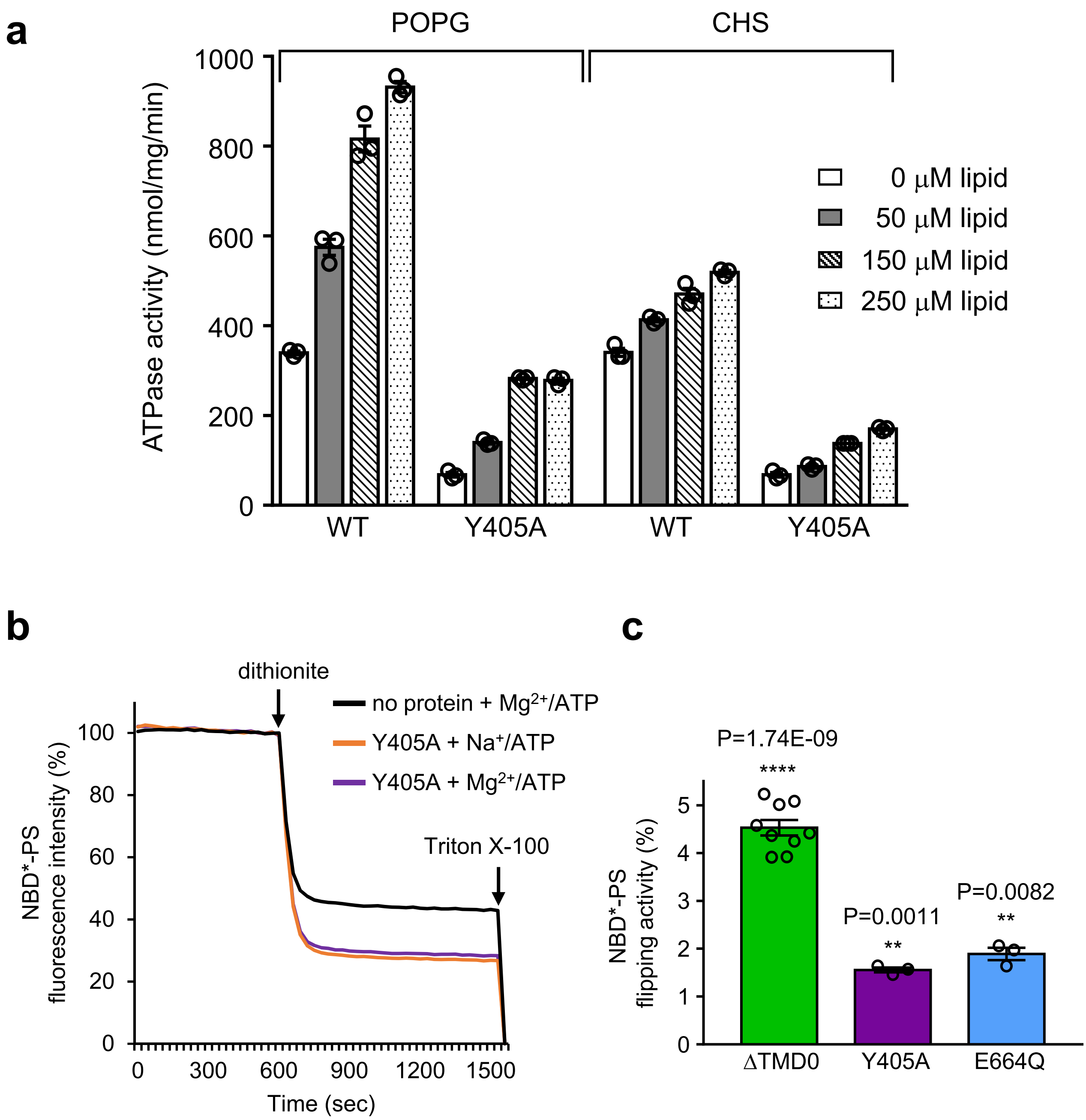

Supplementary Figure 8. Functional characterization of the Y405A mutant. (a) The ATPase activities of DM-purified WT and Y405A mutant were measured by adding various concentrations of POPS or CHS to the reaction mixtures. The results showed that the Y405A mutant had significantly reduced basal and lipid-stimulated ATPase activity. Data for the wild-type are the same as in Figure 1d. Each point is the mean of three replicate experiments using the same preparation. Error bars represent SEM (± standard error of the means). Source data is provided as source data file. (b) Fluorescence traces from NBD*-PS (18:1-16:0) floppase assays of Y405A-containing liposomes in the presence of Mg^{2+}/ATP or Na^{+}/ATP. Protein-free liposomes were used as a negative control. Results shown are averages of three measurements from the same batch of liposome preparation. Source data is provided as source data file. (c) Net percentage of NBD*-PS transported from the outer to the inner leaflet by the Y405A mutant. Data for the wild-type and E664Q are the same as in Figure 2e. Results show the middle value of three separate measurements from the same batch of liposome preparation. Errors represent the standard error of the mean (SEM). The mark ** or **** represents the significant difference ($p < 0.01$ or $p < 0.0001$) between the percentages of NBD*-lipid fluorescence measured in the presence of Mg^{2+}/ATP and Na^{+}/ATP in each assay. The P value was calculated by a two-sided unpaired *t*-test and adjusted using the Welch's correction method. Source data is provided as source data file.

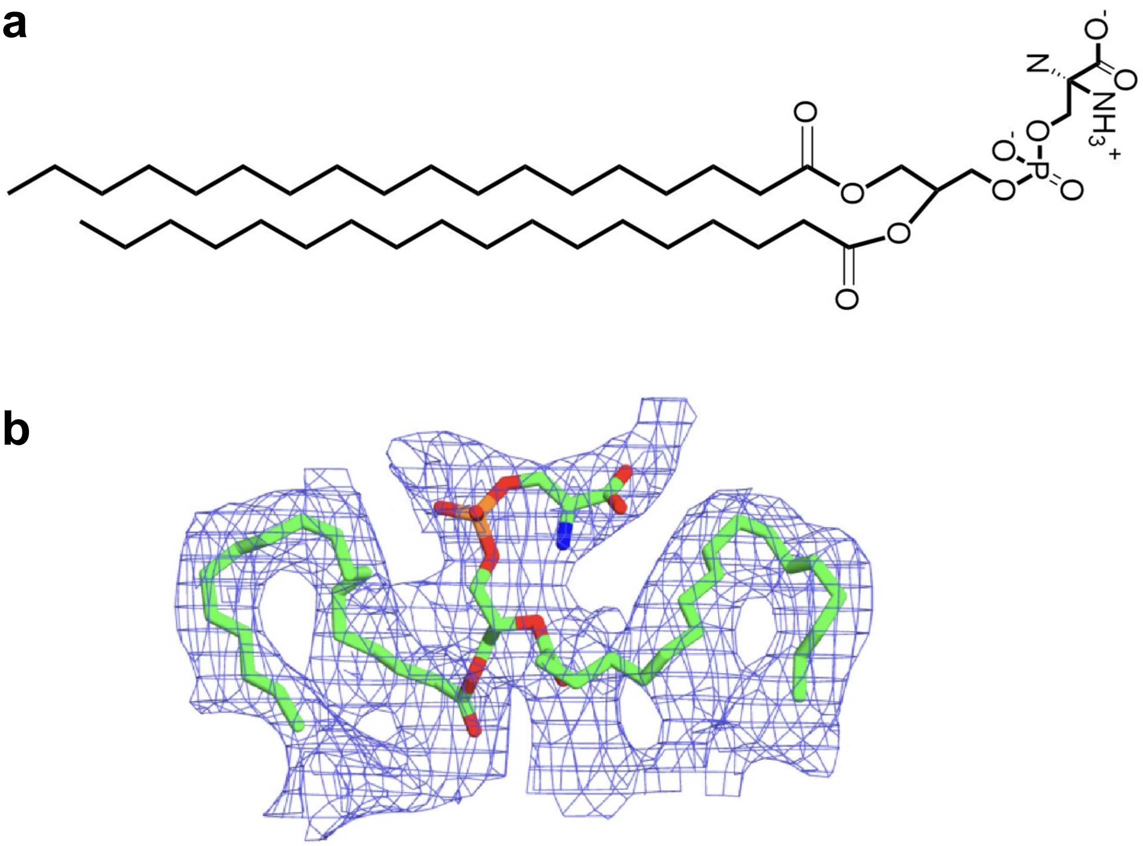

Supplementary Figure 9. Electron density map of 1,2-distearoyl-sn-glycero-3-phosphoserine (18:0-18:0 PS). (a) The chemical structure of the PS. (b) The EM density of bound PS (3 σ level).

a

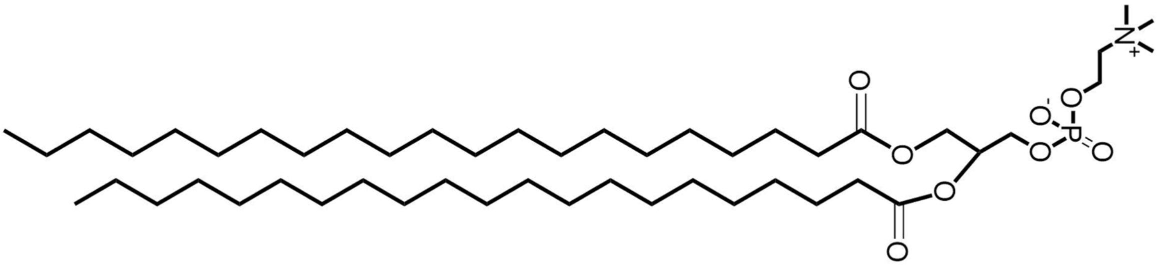

b

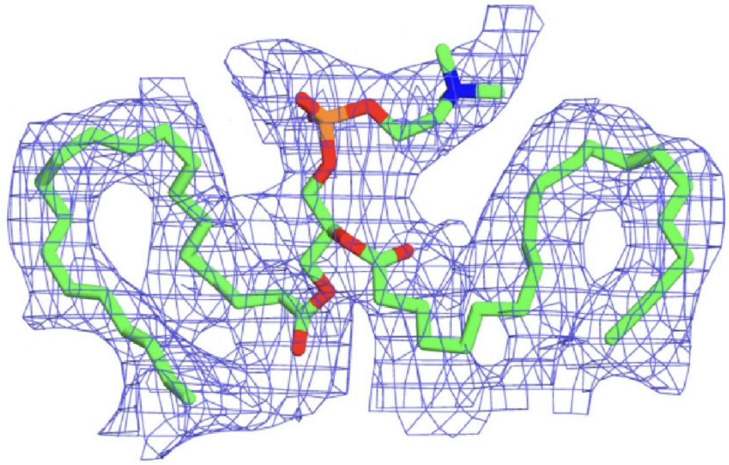

Supplementary Figure 10. Electron density map of 1,2-diheneicosanoyl-sn-glycero-3-phosphocholine (21:0-21:0 PC). (a) The chemical structure of the PC. (b) The EM density of bound PC (3 σ level).

a

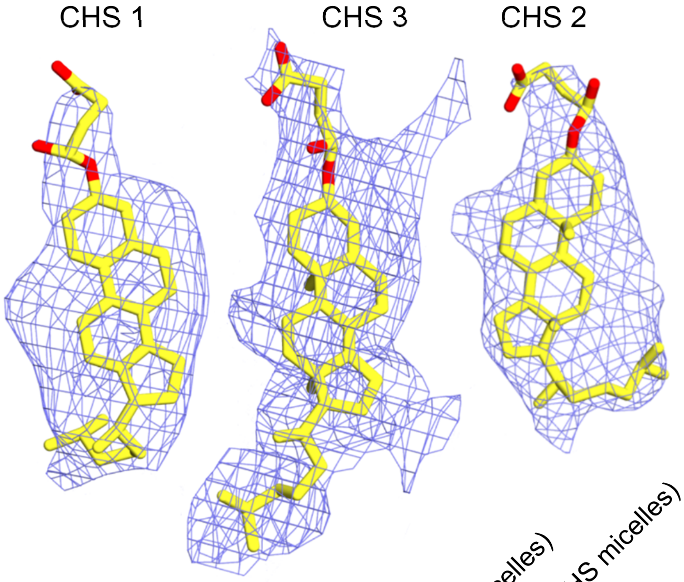

b

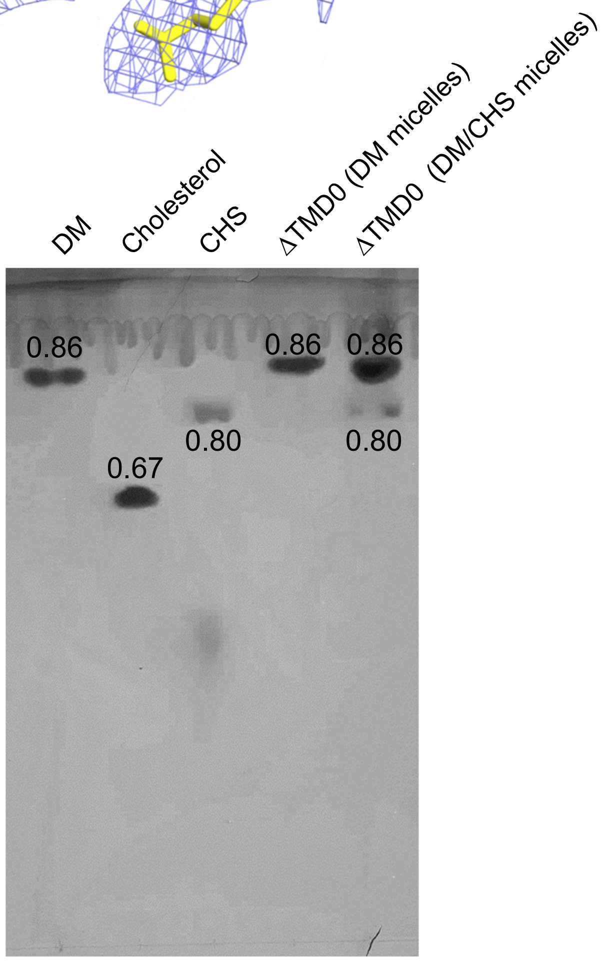

Supplementary Figure 11. CHS binding suggested by cryo-EM maps and TLC analysis obtained from DM/CHS-purified ΔTMD0. (a) EM densities of CHSs are shown at the 3.5 σ level. (b) Identification of lipid species bound to ΔTMD0 by thin layer chromatography (TLC). The Retention factor (Rf) of each spot is indicated.

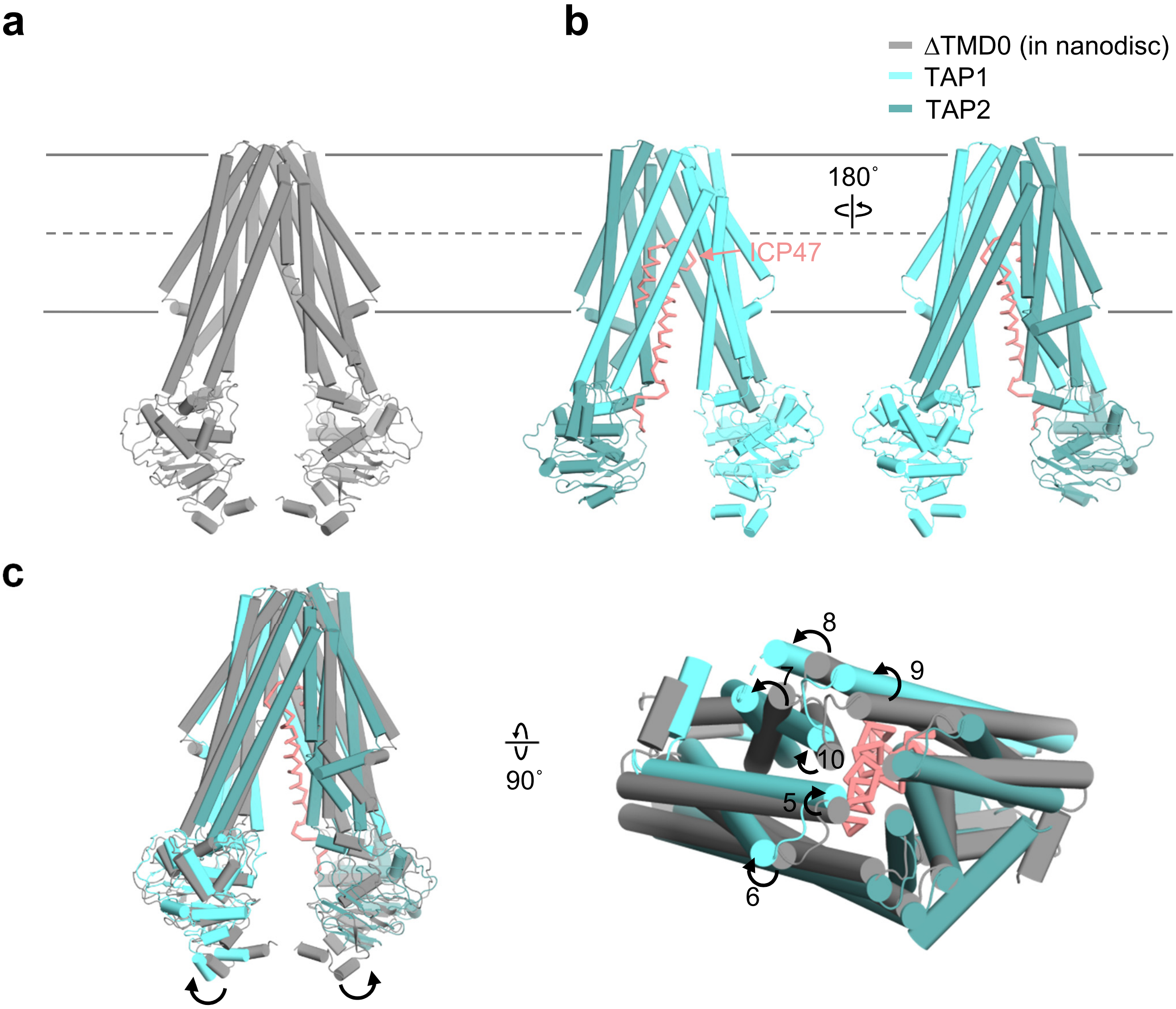

d

```
                        TM7                  TM8
                        298                  343
human TAPL   251 TMVSDLVSQNINVFLRN--IMMVSNIYGKYYKRLSK 355
mouse TAPL   247 TMVSDLVSQNINIFLRN--IMMVSNIYGKYYKRLSK 351
human TAP1   250 STLSDSLSENLSLFLWY--LFLLPKKVGKWYQLLEV 354
human TAP2   215 TLMSNWLPLNANVLLRS--TIAAEKVYNTRHQEVLR 319
                 : :*: :  * .::*     :   :  .. :: :

                        TM9                      TM10
                        405                  449 452 456 459  464 468
human TAPL   371 LNRKEAAAYMYYVWGSG--LIAFIIYEFVLGDCMESVGSVYSGLMQGVGAAEKVF 480
Mouse TAPL   367 LNRKEAAAYMSYVWGSG--LIAFIIYEFVLGDCMESVGSVYSGLMQGVGAAEKVF 476
human TAP1   370 LNQKEAVAYAVNSWTTS--LVTFVLYQMQFTQAVEVLLSIYPRVQKAVGSSEKIF 479
human TAP2   335 LYWRRDLERALYLLVRR--LLSFMIYQESVGSYVQTLVYIYGDMLSNVGAAEKVF 444
                 *  :.              *::*::*:  . . :: :  :*  : . **::**:*
```

Supplementary Figure 12. Structural comparison of ΔTMD0 with human TAP1/2 complex. (a-b) Inward-facing structures of (a) ΔTMD0 and (b) human TAP1/2 complex (PDB ID 5U1D). (c) ΔTMD0 is superimposed on the structure of TAP1/2 complex. Conformational changes of the TM helices are marked by arrows. (d) Partial sequence alignment of TAPL and TAP1/2. Amino acids characterized as functionally important in TAPL are shown in red (F449 separates the central and upper cavities), yellow (residues involved in peptide interaction) and cyan (glycine clusters near the lateral gate).

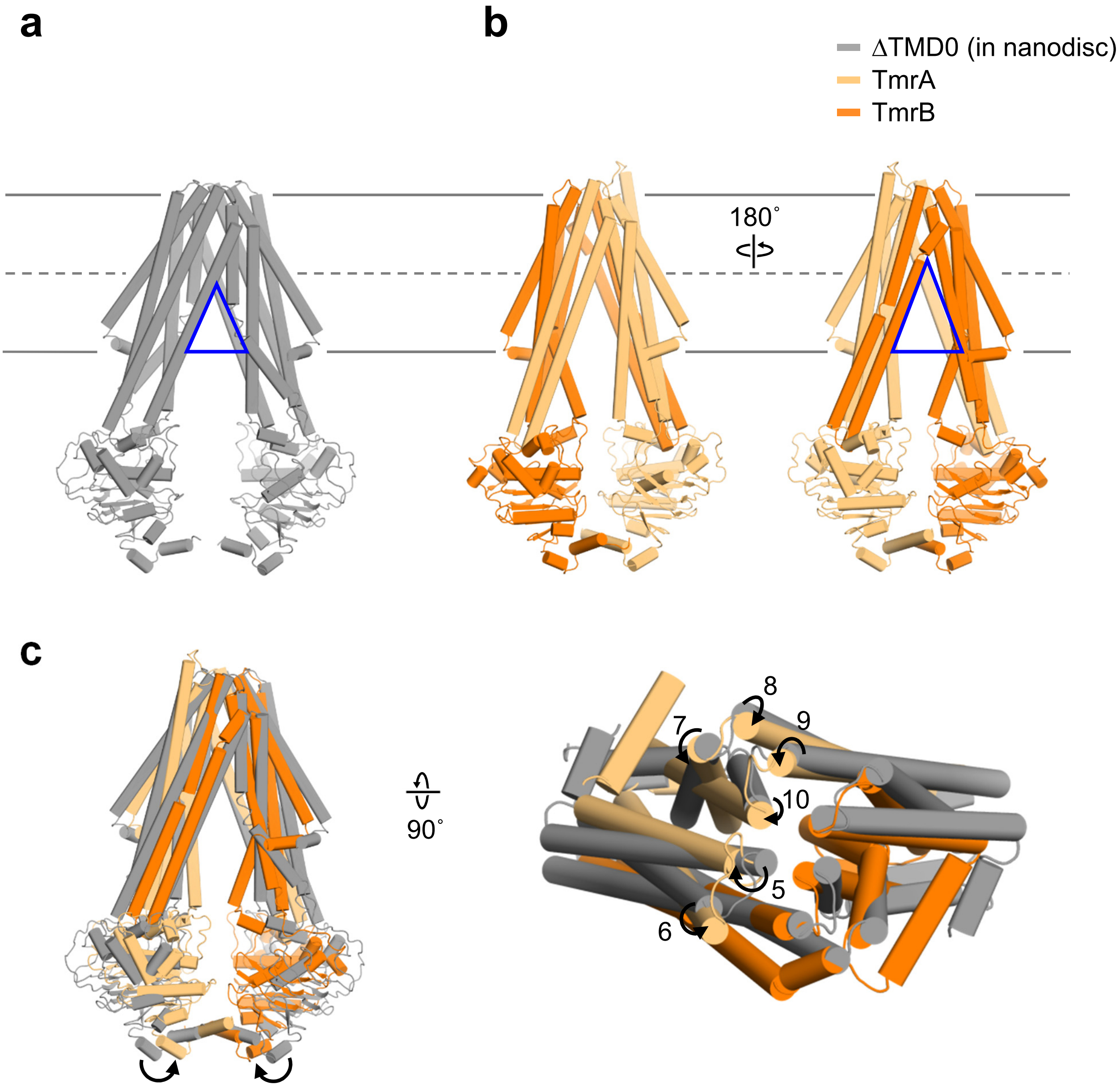

Supplementary Figure 13. Structural comparison of ΔTMD0 with its bacterial homolog TmrAB. (a-b) Inward-facing structures of (a) ΔTMD0 and (b) *T. thermophiles* TmrAB (PDB ID 5MKK). Possible lateral openings in ΔTMD0 and TmrB are indicated by blue triangles. (c) ΔTMD0 superimposed on TmrAB. Conformational changes of the TM helices are marked by arrows.

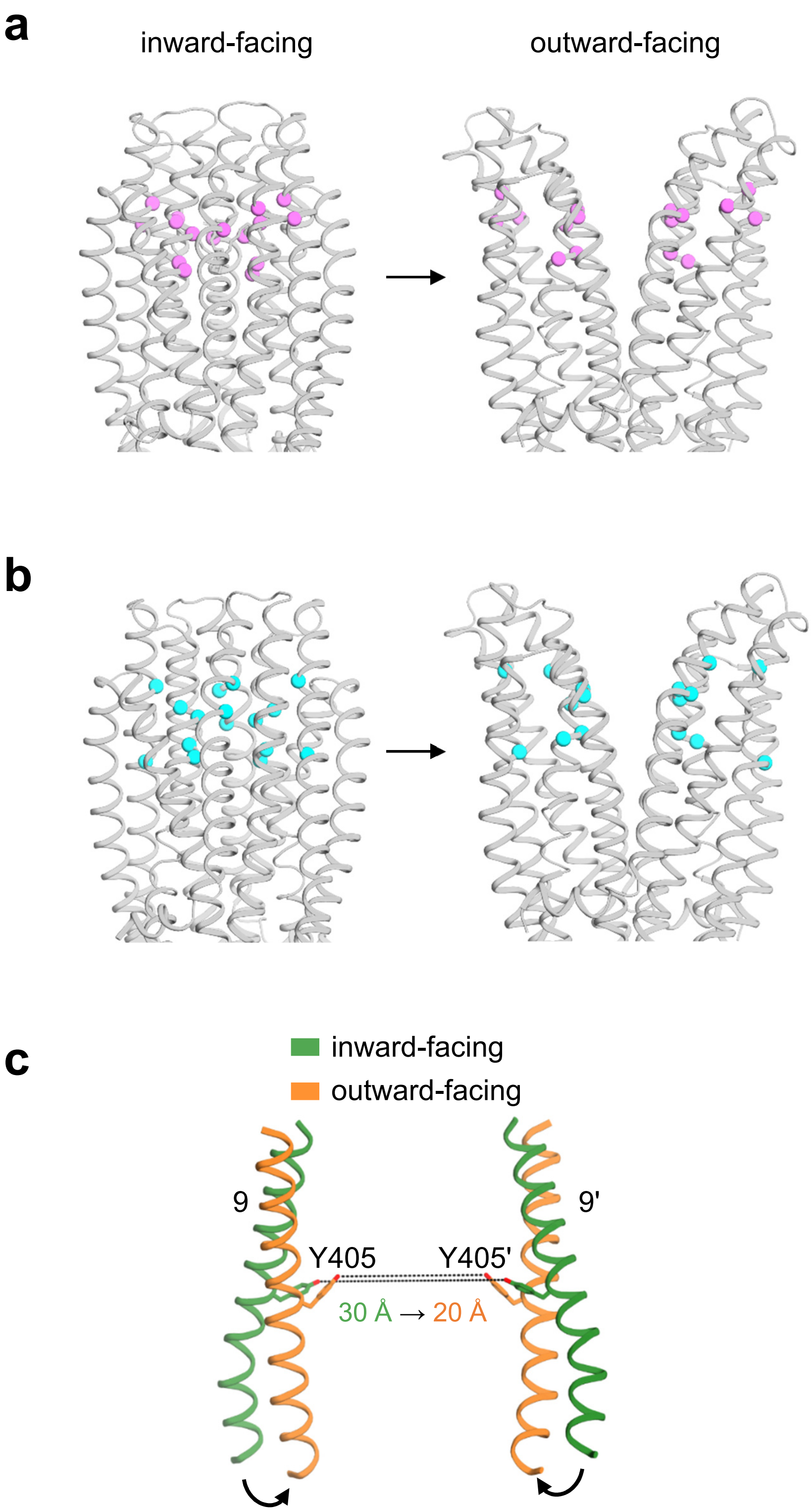

Supplementary Figure 14. Structural transition facilitates release of the substrates by reducing binding affinities. (a) The PG-interacting residues in the inward-facing and the outward-facing state are shown as pink spheres. (b) The CHS-interacting residues in the inward-facing and the outward-facing state are shown as cyan spheres. (c) Superposition of TM9 of the inward-facing (peptide-bound) and outward-facing (ADP·BeF_3-bound) structures of ΔTMD0. Y405 residues are shown as sticks. The view is rotated by 60 degrees along the vertical axis from Fig. 3B.

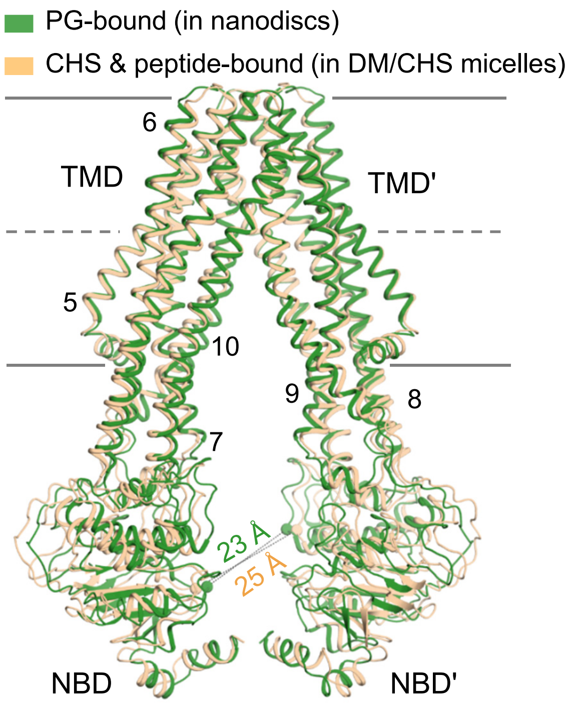

Supplementary Figure 15. The overall folding of ΔTMD0 and the degree of NBD separation are very similar regardless of peptide binding. The structures of PG-bound ΔTMD0 (in nanodiscs) and CHS- and peptide-bound ΔTMD0 (in DM/CHS micelles) are aligned. The Cα distance between the conserved glycine residue (G538) of the Walker A motif of one NBD and the serine residue (S640) of the signature motif of the other is indicated.

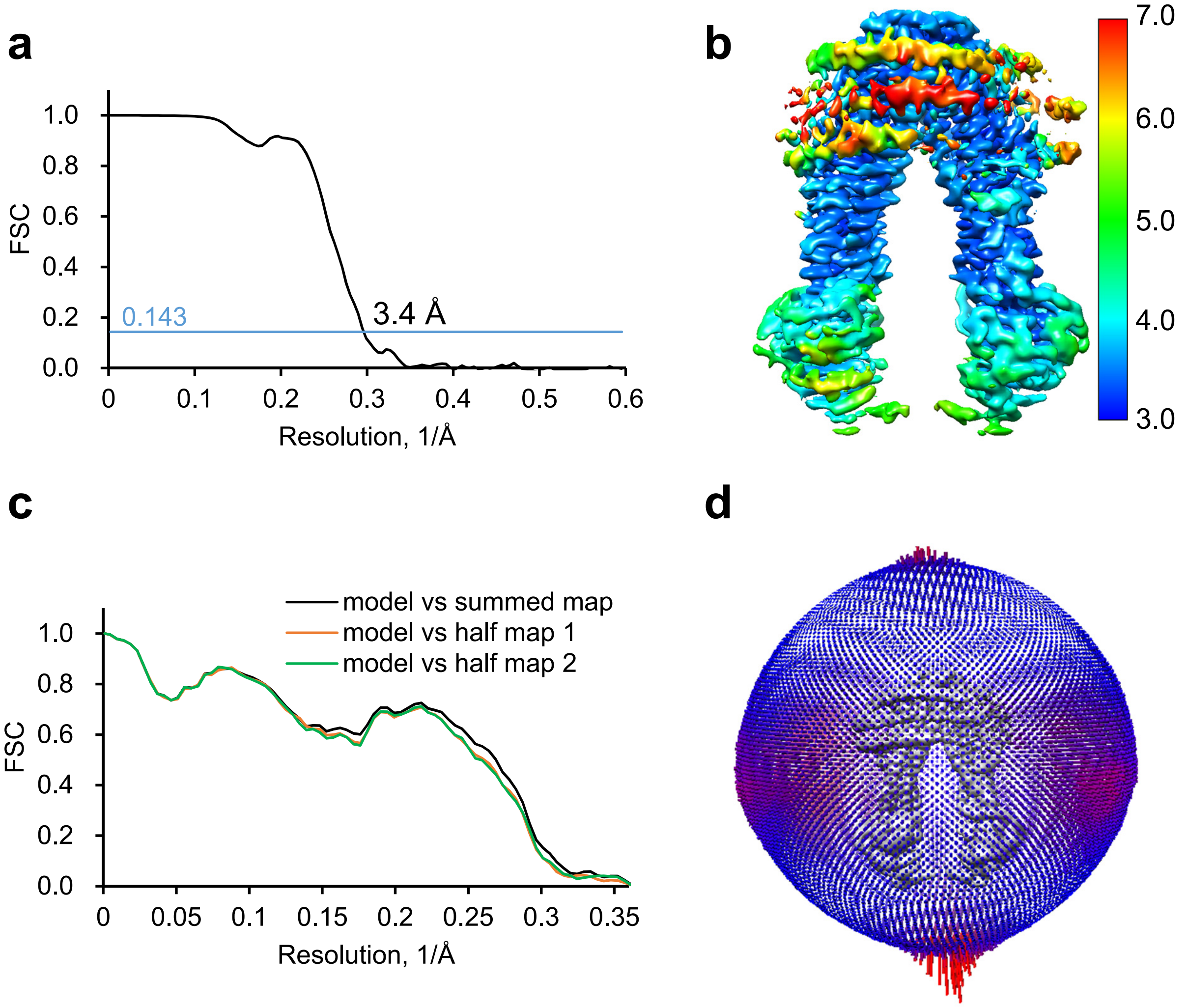

Supplementary Figure 16. Cryo-EM analysis and refinement of the atomic model of inward-facing ΔTMD0 (PG-bound). (a) The Fourier Shell Correlation (FSC) curve. The blue line represents the 0.143 FSC cutoff criterion. (b) Local resolution map calculated with cryoSPARC. (c) FSC curves for cross validation between the refined model and the map calculated from the full dataset, the half map used for refinement (work) and the other half map (free). (d) Euler angle distribution of the refined particles.

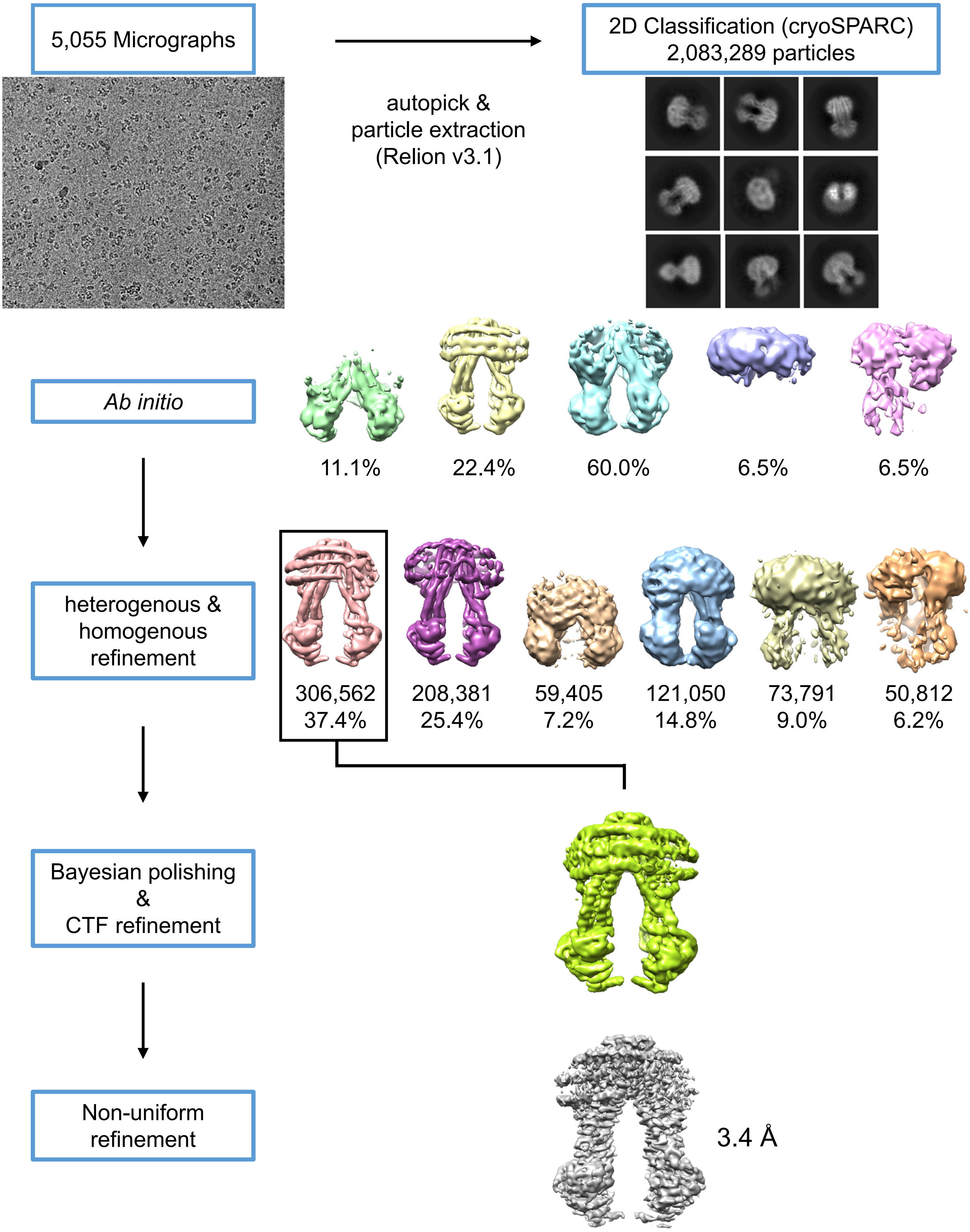

Supplementary Figure 17. Flowchart of cryo-EM processing of data for inward-facing ΔTMD0 (PG-bound). Overview of the cryo-EM data processing pipeline. A representative micrograph (drift-corrected and dose-weighted) and 2D class averages of ΔTMD0 particles are also shown.

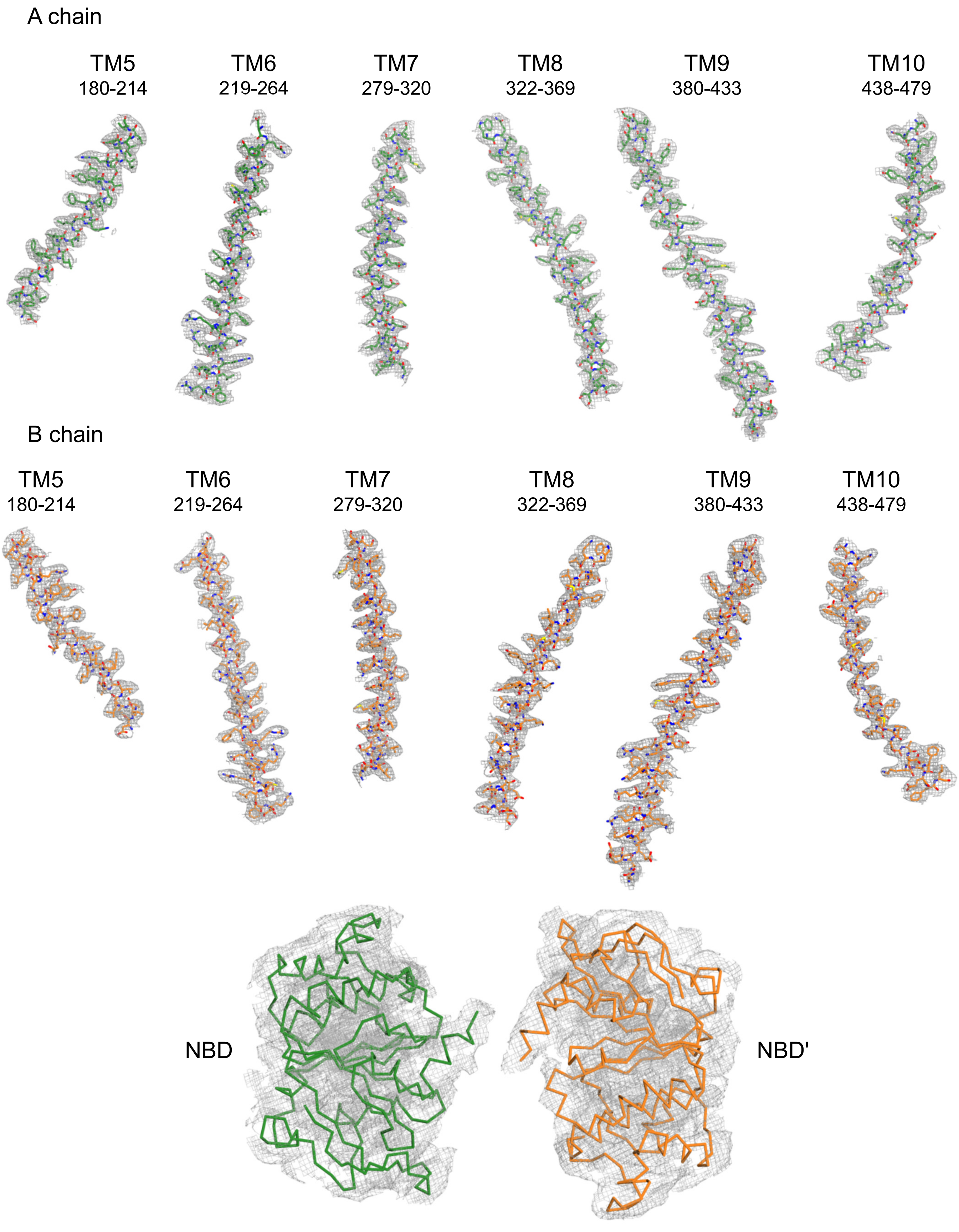

Supplementary Figure 18. EM density maps of the inward-facing ΔTMD0 (PG-bound). EM densities corresponding to each TM helix and two NBD domains are shown.

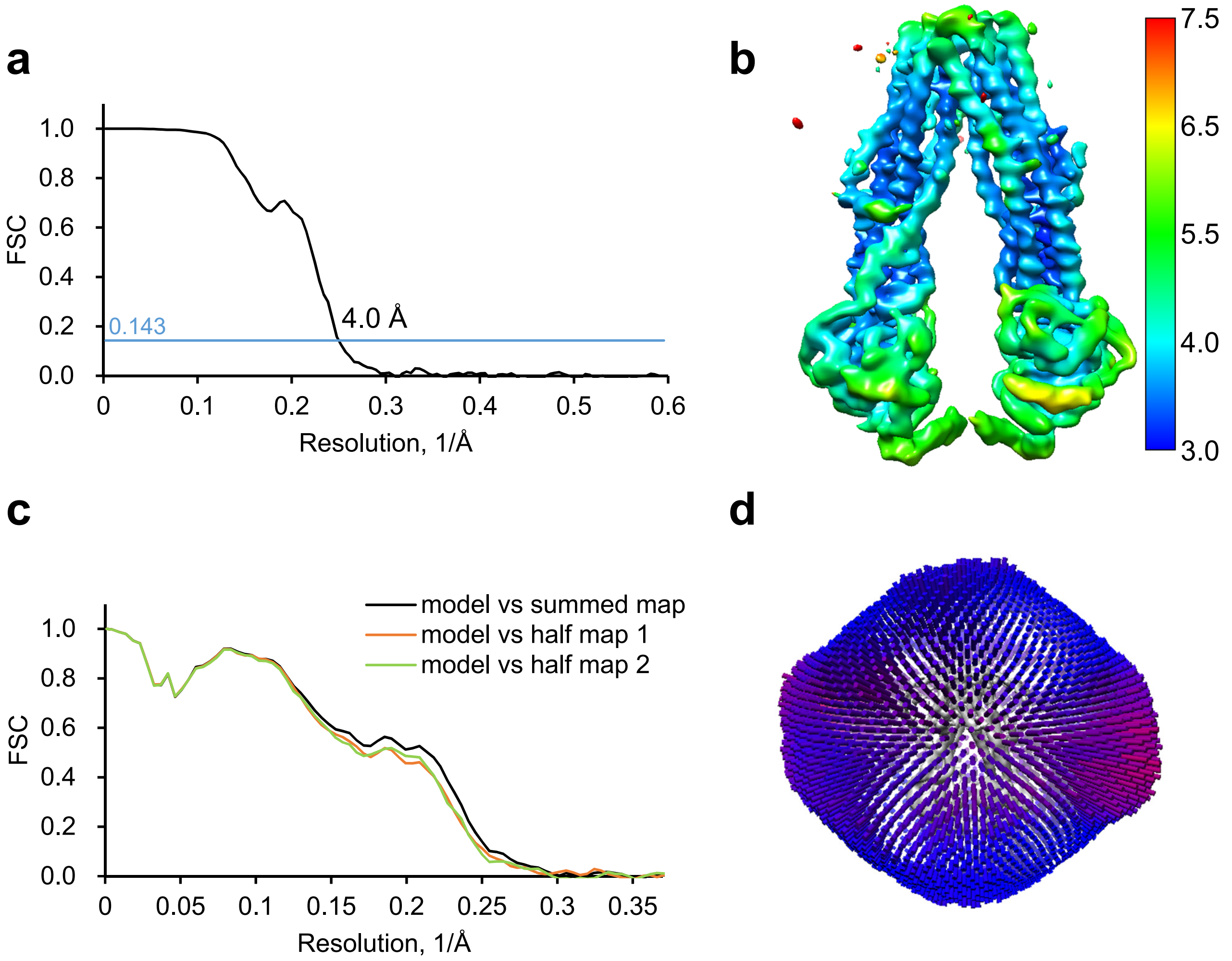

Supplementary Figure 19. Cryo-EM analysis and refinement of the atomic model of peptide-bound ΔTMD0. (a) Fourier Shell Correlation (FSC) curve. The blue line represents the 0.143 FSC cutoff criterion. (b) Local resolution map calculated by cryoSPARC. (c) FSC curves for cross validation between the refined model and the map calculated from the full dataset, the half map used for refinement (work) and the other half map (free). (d) Euler angle distribution of the refined particles.

.

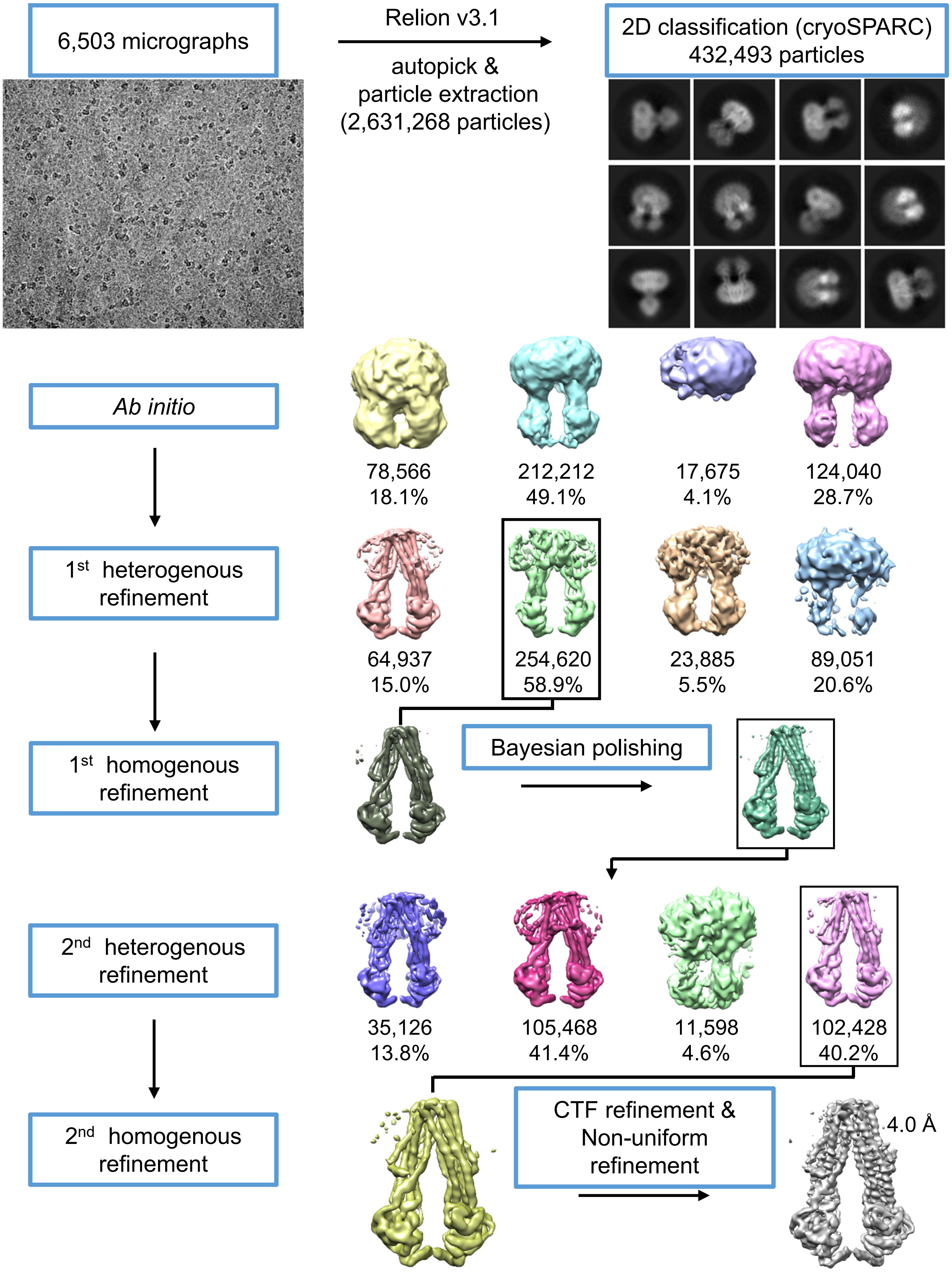

Supplementary Figure 20. Cryo-EM data-processing flowchart of peptide-bound ΔTMD0. Overview of the cryo-EM data-processing pipeline. A representative micrograph (drift-corrected and dose-weighted), and 2D class averages of ΔTMD0 particles are also shown.

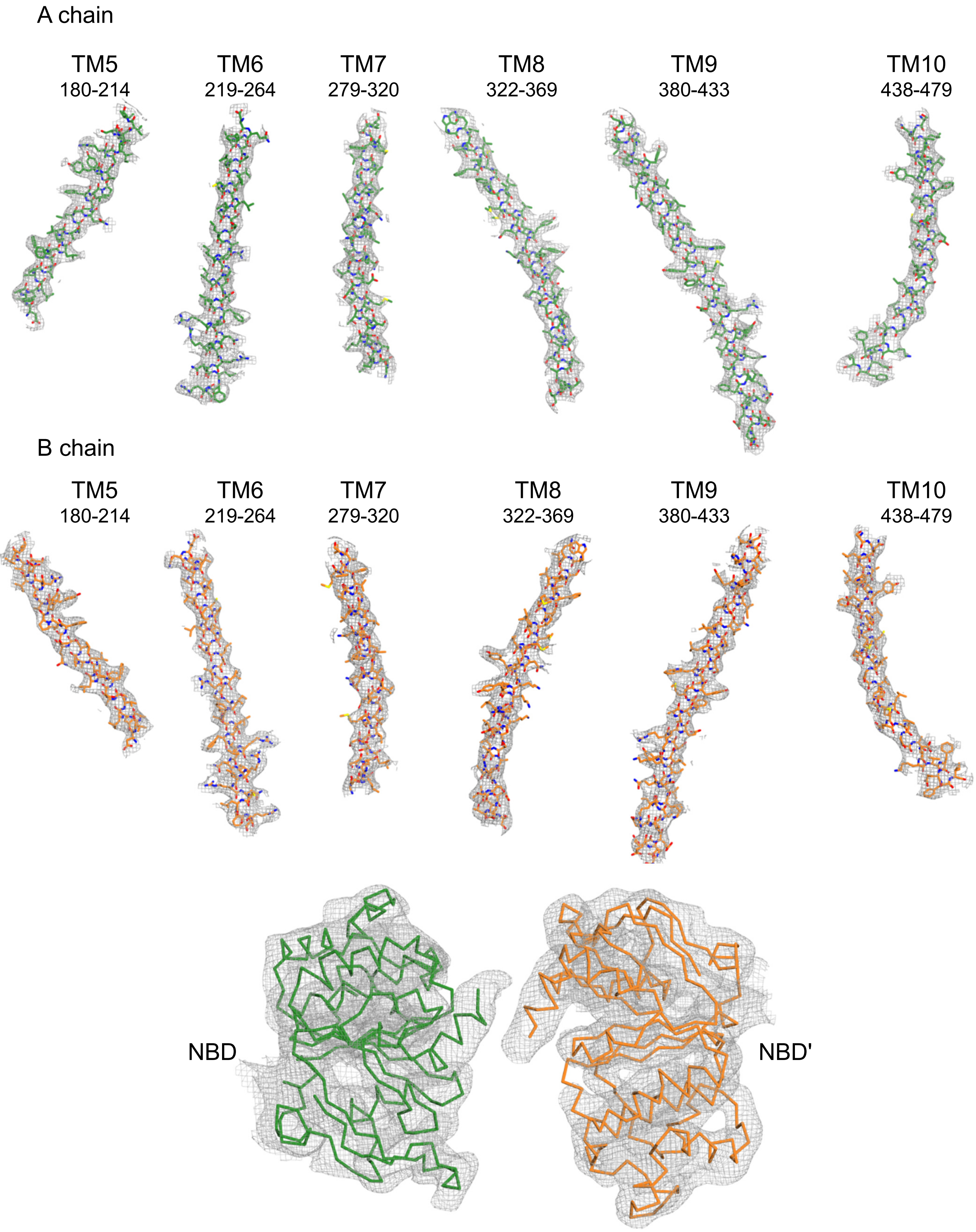

Supplementary Figure 21. EM density maps of the TM helices and NBDs of peptide-bound ΔTMD0.
EM densities corresponding to each TM helix and two NBD domains are shown.

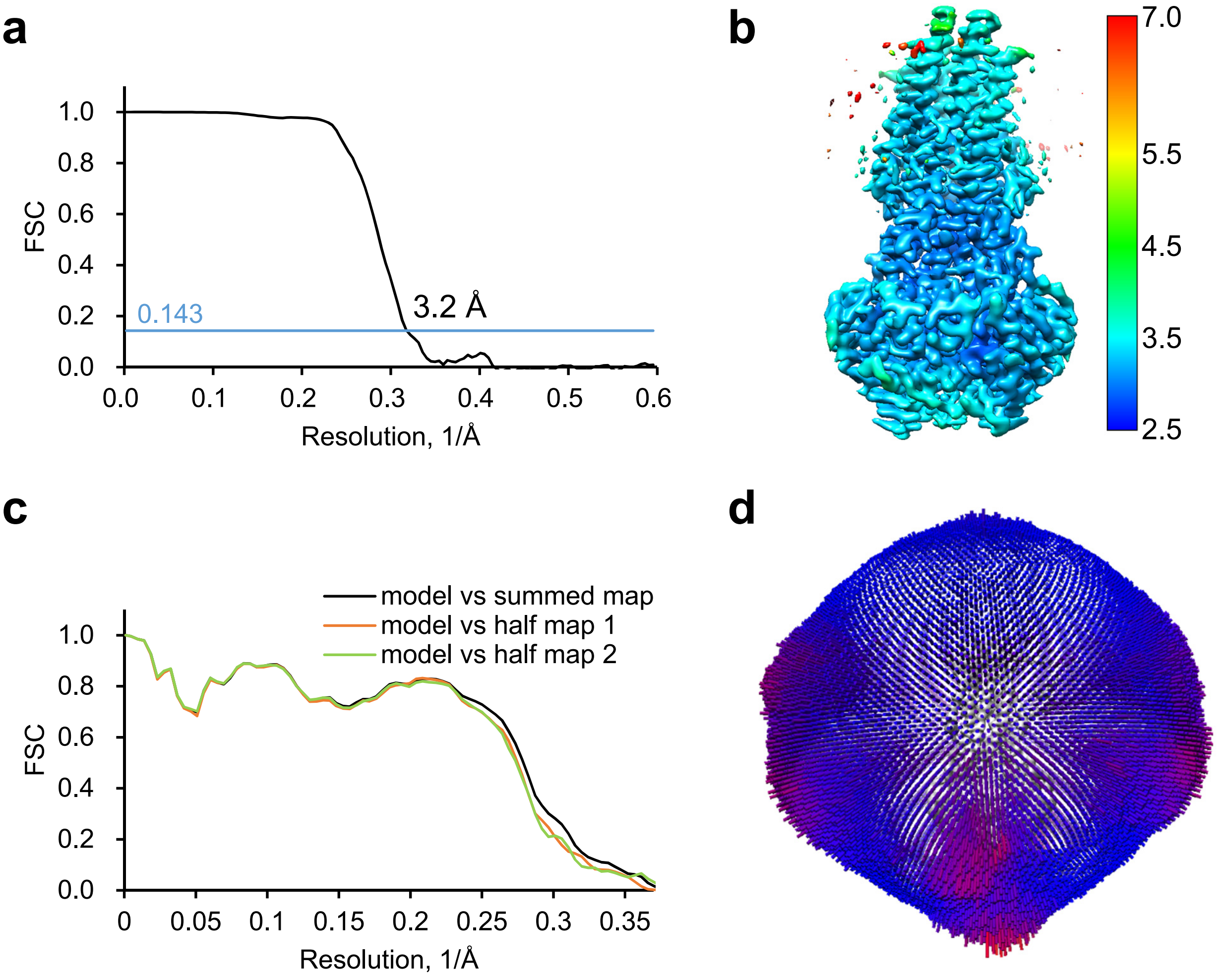

Supplementary Figure 22. Flowchart of cryo-EM processing of data for outward-facing ΔTMD0 (ADP·BeF$_3$-bound). (a) The Fourier Shell Correlation (FSC) curve. The blue line represents the 0.143 FSC cutoff criterion. (b) Local resolution map calculated with cryoSPARC. (c) FSC curves for cross validation between the refined model and the map calculated from the full dataset, the half map used for refinement (work) and the other half map (free). (d) Euler angle distribution of the refined particles.

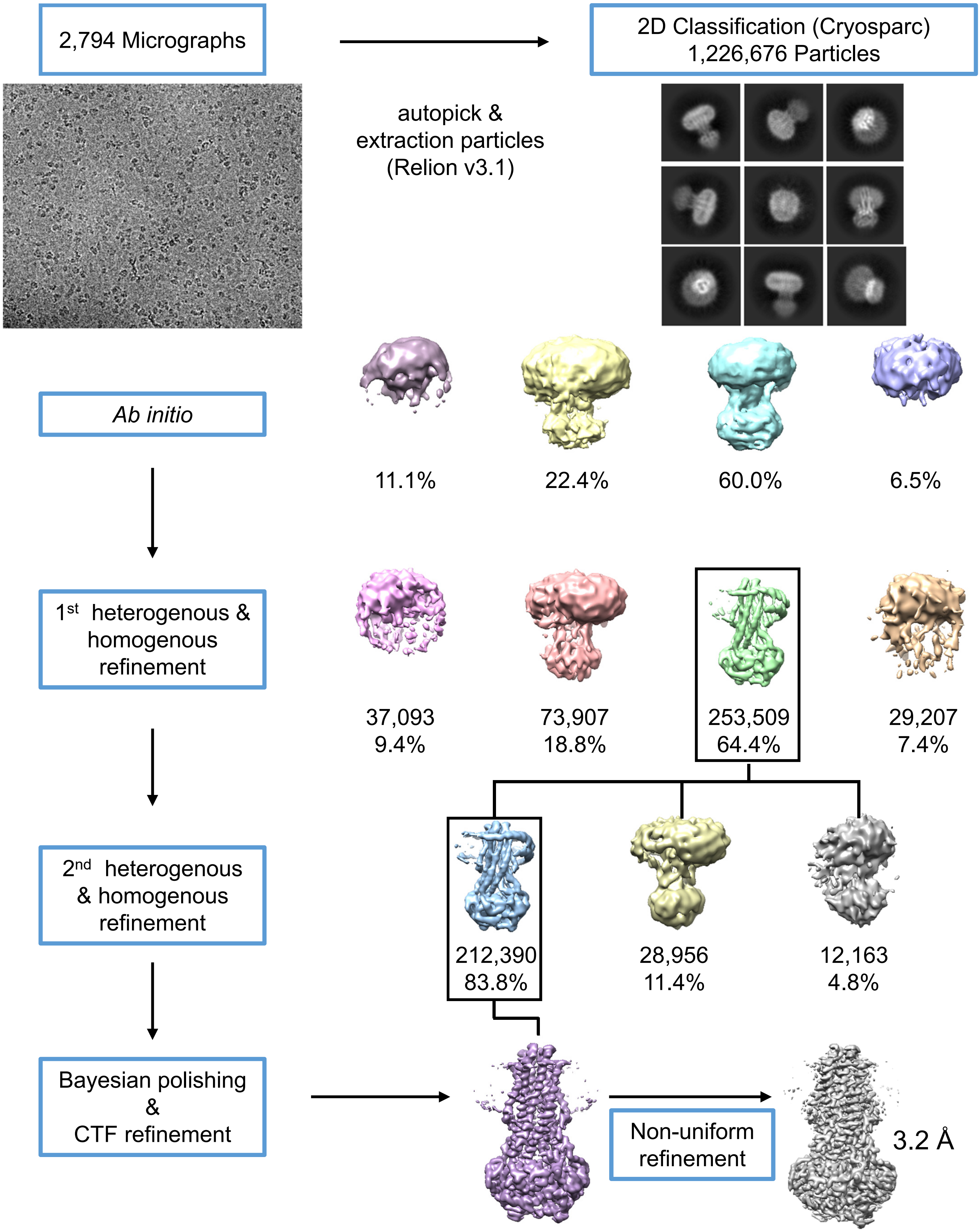

Supplementary Figure 23. Flowchart of cryo-EM processing of data for outward-facing ΔTMD0 (ADP·BeF$_3$-bound). Overview of the cryo-EM data processing pipeline. A representative micrograph (drift-corrected and dose-weighted) and 2D class averages of ΔTMD0 particles are also shown.

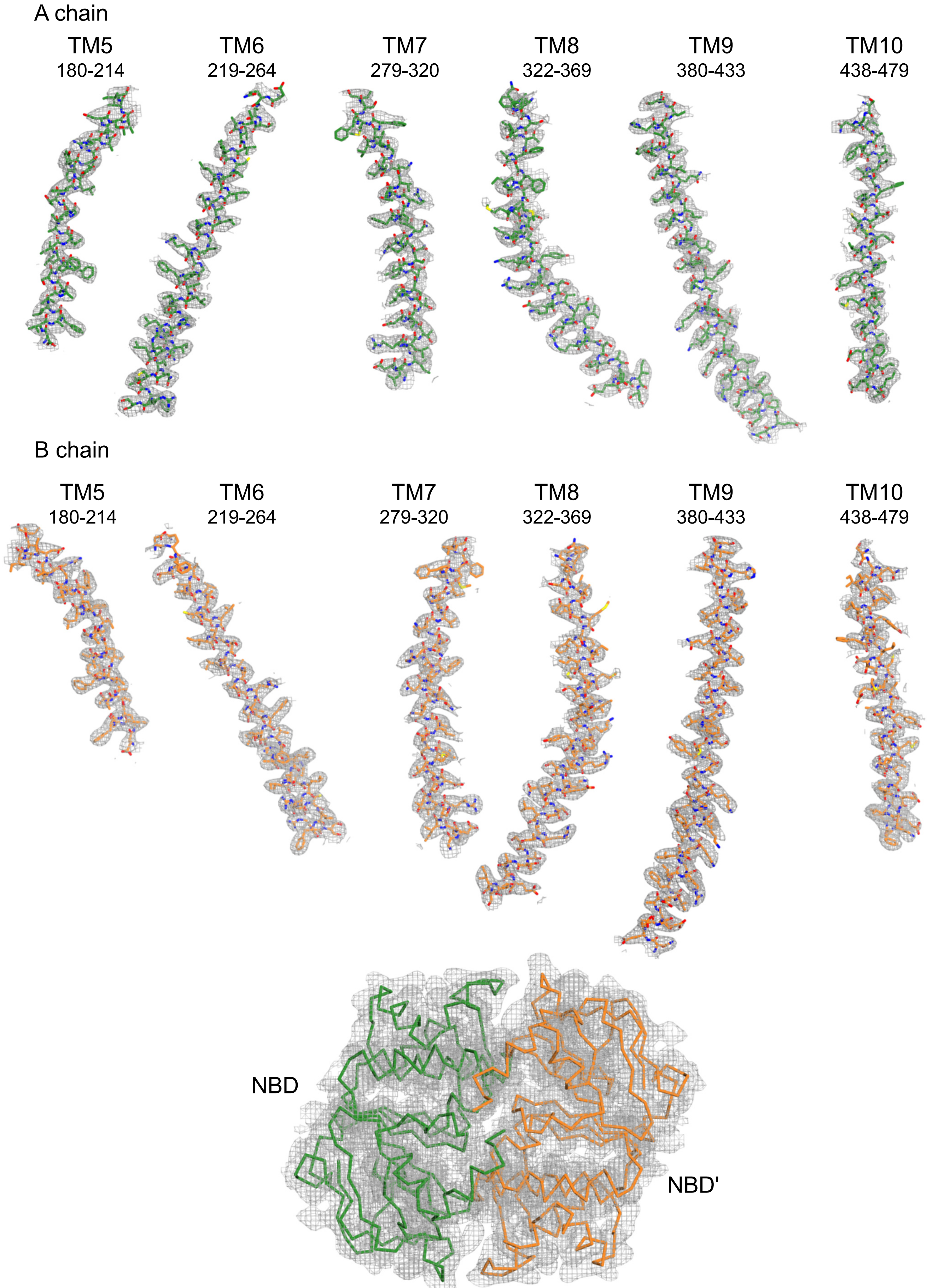

Supplementary Figure 24. EM density maps of the outward-facing ΔTMD0 (ADP·BeF_3-bound). EM densities corresponding to each TM helix and two NBD domains are shown.

Table S1. List of primers used in this study

Mutation site	Forward primer (5'→ 3')	Reverse primer (5'→ 3')
Full-length	AAGTGGATCCATGAGGCTGTGGAAGGCG	TCGGGCGGCCGCAGGCCTTGTGTTCAGTGTT
ΔTMD0	ACTACGGATCCATGGACGCAGAGGCCCTGGAGCC	ACTACGCGGCCGCAGGCCTTGTGTTCAGTGTTGCTG
N301A	TCTCCCAGAACATCGCTATCTTCCTGAGG	CCTCAGGAAGATAGCGATGTTCTGGGAGA
Q298A	CGACCTGGTCTCCGCTAACATCAACATC	GATGTTGATGTTAGCGGAGACCAGGTCG
Y405A	CAGCGGCCGCTATGTCCTA	TAGGACATAGCGGCCGCTGCTT
E456A	GGAGACTGCATGGCTTCCGTGGGC	GCCCACGGAAGCCATGCAGTCTCC
E664Q	GCTTATCCTGGACCAGGCCACCAGTG	CTGGTGGCCTGGTCCAGGATAAG

Table S2. Cryo-EM data collection, refinement and validation statistics

	PG-bound<br>EMDB-31723<br>PDB 7V5D<br>(nanodisc)	CHS & peptide-bound<br>EMDB-31955<br>PDB ID 7VFI<br>(DM/CHS micelles)	ADP·BeF_3-bound<br>EMDB-31722<br>PDB 7V5C<br>(nanodisc)
**Data collection and processing**			
Magnification	X 100,000	X 100,000	X 100,000
Voltage (kV)	200	200	200
Electron exposure (e^-/Å^2)	40	40	40
Defocus range (μm)	-0.8 ~ -2.2	-0.8 ~ -2.2	-0.8 ~ -2.2
Pixel size (Å)	0.83	0.83	0.83
Symmetry imposed	C1	C1	C2
Initial particle images (no.)	2,083,289	2,631,268	1,226,676
Final particle images (no.)	306,562	102,428	212,390
Map resolution (Å)	3.4	4.0	3.2
**Refinement**			
Initial model used (PDB code)	3ZDQ	7V5D	7V5D
Map sharpening B factor (Å^2)	145.6	148.8	137.5
**Model composition**			
Protein residues (non-H)	8,771	8727	8,852
Ligand (non-H)	51	176	64
**B factors (Å^2)**			
Protein	136.64	254.24	101.6
Ligand	94.76	266.75	81.5
**R.m.s. deviations**			
Bond lengths (Å)	0.010	0.006	0.008
Bond angles (°)	0.902	0.902	1.004
**Validation**			
MolProbity score	2.0	1.9	1.8
Clashscore	8.6	9.6	6.6
Poor rotamers (%)	0.6	0.1	0
**Ramachandran plot**			
Favored (%)	91.5	93.4	93.3
Allowed (%)	8.5	6.5	6.6
Disallowed (%)	0	0	0
