# Supplementary material for: The lysosomal transporter TAPL has a dual role as peptide translocator and phosphatidylserine floppase
Source: Nat Commun. 2022 Oct 4;13:5851. doi: 10.1038/s41467-022-33593-2 (PMC9532399; doi:10.1038/s41467-022-33593-2)
Supplement: Supplementary file 3 — Reporting Summary [file 41467_2022_33593_MOESM3_ESM.pdf]

nature portfolio

Corresponding author(s): Mi Sun Jin

Last updated by author(s): Aug 29, 2022

Reporting Summary

Nature Portfolio wishes to improve the reproducibility of the work that we publish. This form provides structure for consistency and transparency in reporting. For further information on Nature Portfolio policies, see our Editorial Policies and the Editorial Policy Checklist.

Statistics

For all statistical analyses, confirm that the following items are present in the figure legend, table legend, main text, or Methods section.

n/a	Confirmed	
☐	☒	The exact sample size (*n*) for each experimental group/condition, given as a discrete number and unit of measurement
☐	☒	A statement on whether measurements were taken from distinct samples or whether the same sample was measured repeatedly
☐	☒	The statistical test(s) used AND whether they are one- or two-sided<br>*Only common tests should be described solely by name; describe more complex techniques in the Methods section.*
☒	☐	A description of all covariates tested
☒	☐	A description of any assumptions or corrections, such as tests of normality and adjustment for multiple comparisons
☐	☒	A full description of the statistical parameters including central tendency (e.g. means) or other basic estimates (e.g. regression coefficient) AND variation (e.g. standard deviation) or associated estimates of uncertainty (e.g. confidence intervals)
☐	☒	For null hypothesis testing, the test statistic (e.g. *F*, *t*, *r*) with confidence intervals, effect sizes, degrees of freedom and *P* value noted<br>*Give P values as exact values whenever suitable.*
☐	☒	For Bayesian analysis, information on the choice of priors and Markov chain Monte Carlo settings
☒	☐	For hierarchical and complex designs, identification of the appropriate level for tests and full reporting of outcomes
☒	☐	Estimates of effect sizes (e.g. Cohen's *d*, Pearson's *r*), indicating how they were calculated

Our web collection on statistics for biologists contains articles on many of the points above.

Software and code

Policy information about availability of computer code

Data collection	Cryo-EM data was collected with the EPU v2.8.0 on the FEI Talos Arctica microscope.
Data analysis	Relion v3.1.0<br>- Motion corrections were performed by using MotionCor2 v1.3.0.<br>- CTFFIND 4 was used for estimating contrast transfer function (CTF).<br>- Manual and Auto particle picking were performed.<br>-Baysian polishing was processed.<br>cryoSPARC v3.1.0<br>- 2D classification, Ab-initio, 3D heterogeneous refinement and 3D homogeneous refinement were perfomed.<br>- Global and local CTF refinement and Non-uniform refinement were done.<br>Model building<br>- UCSF chimera v1.14, PHENIX v1.18.2 and COOT 0.8.9.2 EL were used.<br>- MolProbity v4.5.1 was used for validation of the refined model.<br>- All figures in the manuscript were generated using UCSF Chimera v1.14 and PyMOL v2.3.5.<br>Drawing graph and calculating p-value<br>- GraphPad PRISM 7 was used for drawing figures. Also, p-value was analyzed by unpaired t-test in PRISM 7.<br>Drawing lipid structure<br>- ChemDraw 18.2 was used to fig 6 and supplementary figures 8, 9 and 10.

For manuscripts utilizing custom algorithms or software that are central to the research but not yet described in published literature, software must be made available to editors and reviewers. We strongly encourage code deposition in a community repository (e.g. GitHub). See the Nature Portfolio guidelines for submitting code & software for further information.

Data

Policy information about availability of data

All manuscripts must include a data availability statement. This statement should provide the following information, where applicable:

- Accession codes, unique identifiers, or web links for publicly available datasets
- A description of any restrictions on data availability
- For clinical datasets or third party data, please ensure that the statement adheres to our policy

The atomic coordinates of the three structures have been deposited in the Protein Data Bank with accession codes 7V5D [http://doi.org/10.2210/pdb7V5D/pdb] (PG-bound), 7VFI [http://doi.org/10.2210/pdb7VFI/pdb] (both CHS- and peptide-bound) and 7V5C [http://doi.org/10.2210/pdb7V5C/pdb] (ADP·BeF3-bound). The cryo-EM density maps have been deposited in the Electron Microscopy Data Bank with accession codes EMD-31723 [http://www.ebi.ac.uk/pdbe/entry/ebdb/EMD-31723] (PG-bound), EMD-31955 [http://www.ebi.ac.uk/pdbe/entry/ebdb/EMD-31955] (both CHS- and peptide-bound) and EMD-31722 [http://www.ebi.ac.uk/pdbe/entry/ebdb/EMD-31722] (ADP·BeF3-bound). All of raw data are available from corresponding author upon reasonable request.

Field-specific reporting

Please select the one below that is the best fit for your research. If you are not sure, read the appropriate sections before making your selection.

☒ Life sciences ☐ Behavioural & social sciences ☐ Ecological, evolutionary & environmental sciences

For a reference copy of the document with all sections, see nature.com/documents/nr-reporting-summary-flat.pdf

Life sciences study design

All studies must disclose on these points even when the disclosure is negative.

Sample size	The EM data processing workflow is presented in Supplementary Figs. 16-24. The three datasets with 5,055, 6,503 and 2,794 micrographs were collected for the PG-bound, both CHS- and peptide-bound and ADP·BeF3-bound conformations, respectively. The final particles in each dataset were 306,562, 102,428, 212,390, respectively.
Data exclusions	Micrographs that were estimated low CTF were excluded.
Replication	Three cryo-EM structures were obtained by repeating the experiments and processing each cryo-EM dataset using Relion and cryoSPARC.
Randomization	By using random particle subset in Relion and cryoSPARC, 3D auto refinement was performed and the Fourier shell correction (0.143) was calculated.
Blinding	Blinding was not applicable. Because, particle assignment and resolution estimation were automatically processed by Relion and cryoSPARC.

Reporting for specific materials, systems and methods

We require information from authors about some types of materials, experimental systems and methods used in many studies. Here, indicate whether each material, system or method listed is relevant to your study. If you are not sure if a list item applies to your research, read the appropriate section before selecting a response.

Materials & experimental systems

n/a	Involved in the study	
☒	☐	Antibodies
☐	☒	Eukaryotic cell lines
☒	☐	Palaeontology and archaeology
☒	☐	Animals and other organisms
☒	☐	Human research participants
☒	☐	Clinical data
☒	☐	Dual use research of concern

Methods

n/a	Involved in the study	
☒	☐	ChIP-seq
☒	☐	Flow cytometry
☒	☐	MRI-based neuroimaging

Eukaryotic cell lines

Policy information about cell lines

Cell line source(s)	Sf9 cells (purchased from Expression systems) and Hi5 cells (purchased from Expression systems)
Authentication	None of the cell lines used were authenticated.
Mycoplasma contamination	Cell lines were not tested for mycoplasma contamination.
Commonly misidentified lines (See ICLAC register)	No commonly misidentified cell lines were used.
